# Integration of prolapsing technique and one-stitch method of ileostomy during laparoscopic low anterior resection for rectal cancer: a retrospective study

**DOI:** 10.3389/fsurg.2023.1193265

**Published:** 2023-05-31

**Authors:** Xiangmin Li, Min Tian, Jingbo Chen, Yulin Liu, Hu Tian

**Affiliations:** ^1^ Department of General Surgery, Shandong Provincial Qianfoshan Hospital, Shandong University, Jinan, China; ^2^ Department of Nursing, Shandong Provincial Qianfoshan Hospital, Shandong University, Jinan, China

**Keywords:** prolapsing technique, ileostomy, rectal cancer, natural orifice specimen extraction surgery, laparoscopy

## Abstract

**Background:**

Prolapsing technique is a type of natural orifice specimen extraction surgery that can overcome the difficulty of precise transection of the distal rectum and subsequent anastomosis in a narrow pelvic space. Currently, protective ileostomy is widely utilized in low anterior resection for low rectal cancer, which may reduce the severe consequences caused by anastomotic leakage. The study aimed to combine the prolapsing technique with a one-stitch method of ileostomy and evaluate the surgical outcomes.

**Methods:**

A retrospective analysis was conducted on patients with low rectal cancer who underwent protective loop ileostomy in laparoscopic low anterior resection between January 2019 and December 2022. The patients were divided into prolapsing technique combined with the one-stitch method of ileostomy (PO) group and traditional method (TM) group, and the intraoperative details and early postoperative outcomes of the two groups were measured.

**Results:**

A total of 70 patients met the inclusion criteria, including 30 patients who underwent PO and 40 patients who underwent the traditional procedure. The PO group had a shorter total operative time than the TM group (197.8 ± 43.4 vs. 218.3 ± 40.6 min, *P* = 0.047). The time of intestine function recovery in the PO group was shorter than that in the TM group (24.6 ± 3.8 vs. 32.7 ± 5.4 h, *P* < 0.001). Compared with the TM group, the average VAS score was significantly lower in the PO group (*P* < 0.001). The incidence of anastomotic leakage in the PO group was significantly lower than that in the TM group (*P* = 0.034). The operative time of loop ileostomy was 2.0 ± 0.6 min in the PO group, which was significantly less than 15.1 ± 2.9 min in the TM group. Skin irritation was observed in 2 patients in the PO group and 10 patients in the TM group; therefore, there was a significant difference (*P* = 0.044).

**Conclusion:**

This method is safe and feasible, which reduces the technical difficulty and achieves rapid postoperative recovery with few complications.

## Introduction

Low rectal cancer generally refers to rectal cancer whose lower margin is within 5 cm from the dentate line (1). Currently, extra abnormal incision for specimen extraction and double-stapling technique are commonly used in the sphincter-preserving surgery (2). Natural orifice specimen extraction surgery (NOSES) is characterized by the removal of specimen from the natural orifice (anus or vagina), with better cosmetic effect, less postoperative pain, lower risk of incision infection, decreasing incidence of incisional hernia, and shorter length of hospital stay. Transanal prolapsing technique is a type of NOSES. In previous studies of NOSES, there may be risks of abdominal cavity contamination and tumor cell shedding during the process of placement of the anvil of circular stapler in the abdomen and fixation of it to the proximal colonic stump intracorporeally (3). In recent years, diverting ileostomy is widely used to protect high-risk anastomosis. In the conventional ileostomy, a stoma is constructed by using a supporting rod and suturing the intestine's wall transcutaneously or intracutaneously, which is associated with a prolonged duration of operation and a high incidence of stoma-related complications, such as peristomal skin disorders and mucocutaneous separation (4). We describe herein an ideal technique that combined prolapsing technique with the one-stitch method of ileostomy, avoiding abdominal cavity contamination and tumor cell shedding and reducing stoma-related complications, and compare the short-term outcomes with the traditional procedure.

## Methods

### Patient selection and variables

The patients with rectal cancer who underwent laparoscopic low anterior resection and protective loop ileostomy were collected from Shandong Provincial Qianfoshan Hospital between January 2019 and December 2022. The inclusion criteria were as follows: (1) a diagnosis of rectal adenocarcinoma by preoperative colonoscopic pathological examination, (2) the lower margin of the tumor was within 2–5 cm from the dentate line, (3) no intestinal obstruction or perforation, (4) no distant metastasis, (5) T2–T3 tumor on MRI, (6) rectal wall invasion account for less than 1/2 circumference, and (7) body mass index (BMI) of <30 kg/m^2^ (1). The exclusion criteria were as follows: (1) patients who underwent abdominoperineal resection or Hartmann's procedure, (2) radiation therapy or chemotherapy was performed before surgery, (3) the laparoscopic procedure was converted to open surgery, and (4) incomplete clinical and pathological data. According to the procedures of specimen extraction and protective loop ileostomy, all patients were divided into two groups, such the prolapsing technique combined with the one-stitch method of ileostomy (PO) group and the traditional method (TM) group.

The preoperative clinical variables of patients, such as sex, age, BMI, tumor distance from the dentate line, diabetes mellitus, and American Society of Anesthesiologists (ASA) grade, were recorded. The postoperative variables included operation time, estimated intraoperative blood loss, intestine function recovery, postoperative complications, and postoperative pain score. The pathologists determined the pathologic stage of rectal cancer and the quality of the total mesorectal excision (TME). A 10-point visual analog scale (VAS) was used to measure the intensity of the pain, with a high score indicating much pain. The VAS scores were collected on the first three postoperative days. The stoma-associated complications, such as necrosis, bleeding, stricture, retraction, prolapse, mucocutaneous separation, skin irritation, and parastomal hernia, were also investigated. Patients were followed up until ileostomy closure or 6 months after initial surgery. All included patients and their families signed a written informed consent. This study was approved by the hospital’s ethics committee. All operations were performed by the same group of surgeons.

### Surgical procedures

The patient was placed in Lloyd-Davies position under general anesthesia. The pneumoperitoneum (12–14 mmHg) was performed with the five-hole method. The medial-to-lateral approach was used. The retroperitoneum was incised along the right side of the sigmoid mesentery. Toldt's gap was expanded, and the inferior mesenteric artery and vein were divided after clip placement. The rectum was mobilized to the level of the levator ani muscle, if necessary, to the level of the dentate line through the intersphincteric space.

**Figure 1:**
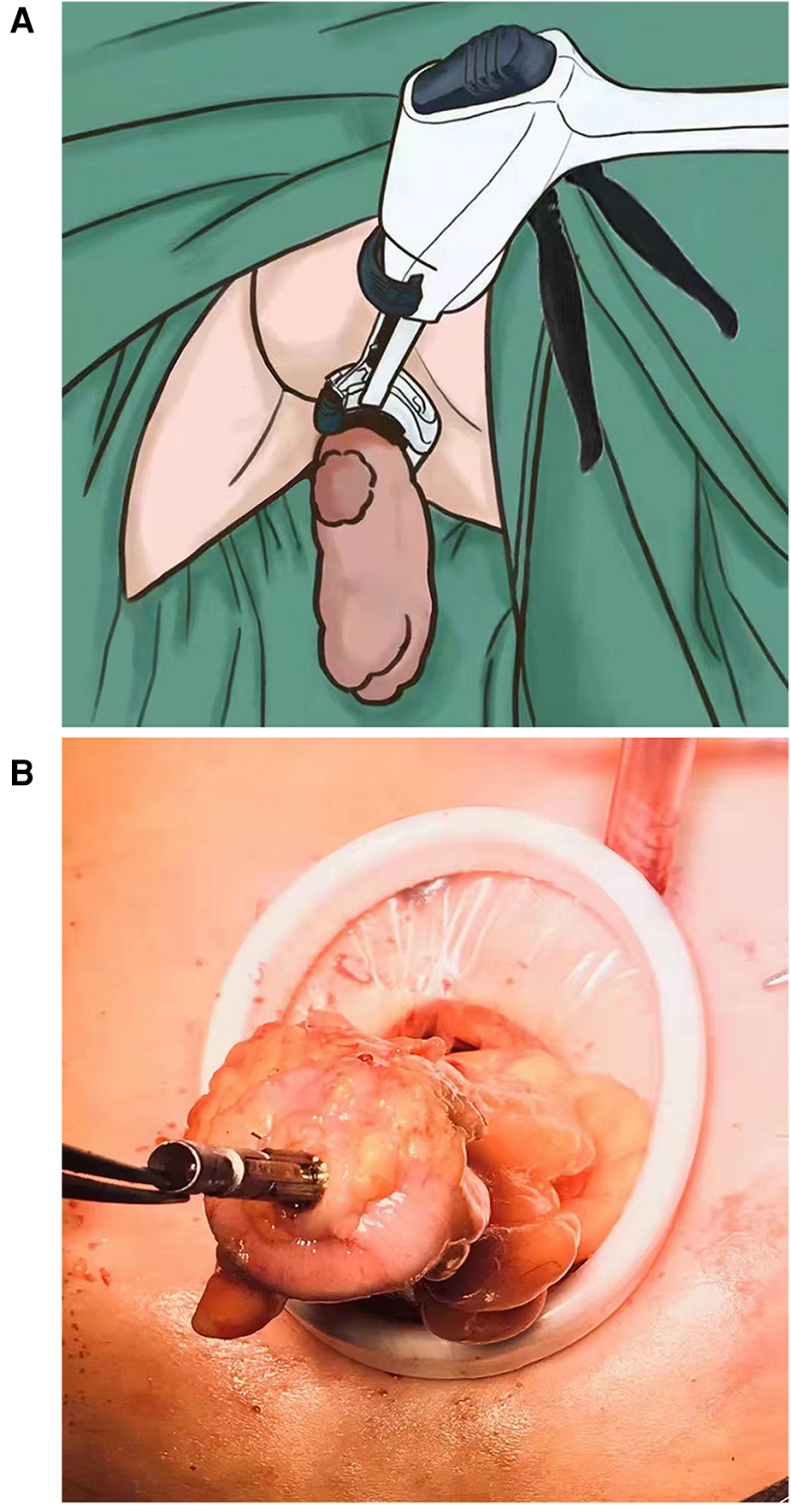
(**A**) Distal rectum was everted and transected under direct vision. (**B**) Anvil of circular stapler was placed in proximal stump and fixed with purse-string suture.

In the PO group, the sigmoid mesentery was fully dissected, and the rectum approximately 10 cm proximal to the tumor was transected using a linear stapler (Endo GIA 60 mm, Covidien, United States). The anus was fully dilated. A sponge forceps was inserted through the anus to grasp the rectal stump under laparoscopic guidance, and the distal rectum was everted out transanally. The rectum was irrigated with diluted povidone-iodine and was then transected 1 cm–2 cm distally from lower margin of the tumor using a stapler (Endo GIA 60 mm, Covidien, United States, or CONTOUR, Ethicon, United States) (Figure 1A). The rectal stump was delivered back to the pelvic cavity.

A 3.5–4 cm longitudinal rectus abdominis muscle-splitting incision was made in the right lower quadrant according to the appropriate stoma position. A wound retractor (small size; HangTian KaDi Technology R & D Institute, Beijing, China) that provided access to the abdominal cavity was attached at the incision. The colonic stump was exteriorized, the edge of it was removed, and purse-string suture was performed. The anvil of the stapler was placed in the proximal colon and fixed after tightening the purse string around the anvil (Figure 1B). Then, the colonic stump was sent back to the abdominal cavity. By covering the wound retractor by a glove, pneumoperitoneum was re-established. End-to-end anastomosis of the colon and rectum was completed using a circular stapler (EEA28, Covidien, United States) that was inserted through the anus.

**Figure 2:**
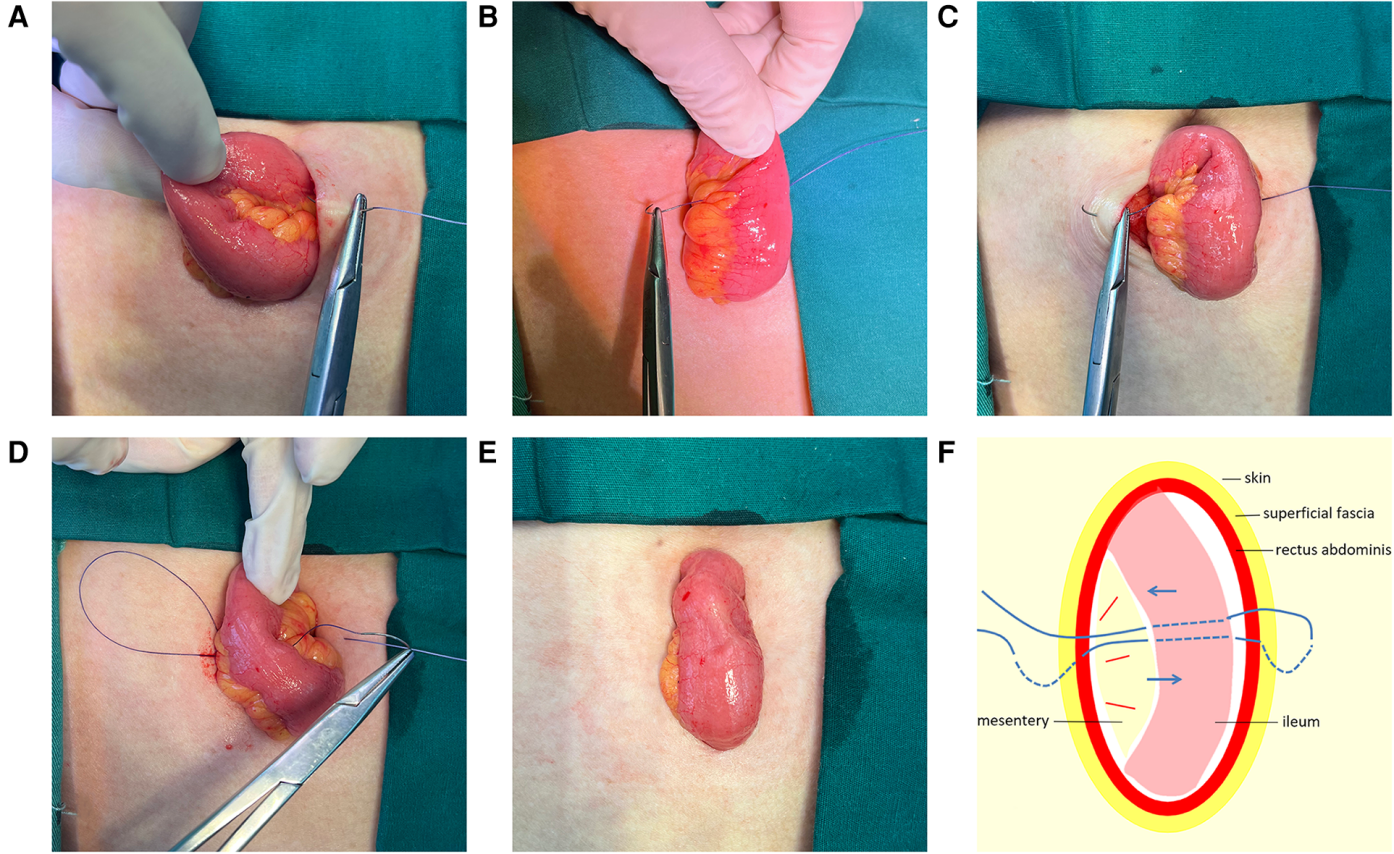
(**A**) Sew into the skin 0.5–1.0 cm away from the incision midpoint at one side of the incision. (**B**) Passed through the avascular area of the ileal mesentery. (**C**) Sew out from the opposite side of the incision at an equal distance. (**D**) Passed through the avascular area of the mesentery to the original side. (**E**) Thread was tightened and knotted to fix the ileal stoma. (**F**) A schematic diagram of the “one-stitch” method.

**Figure 3:**
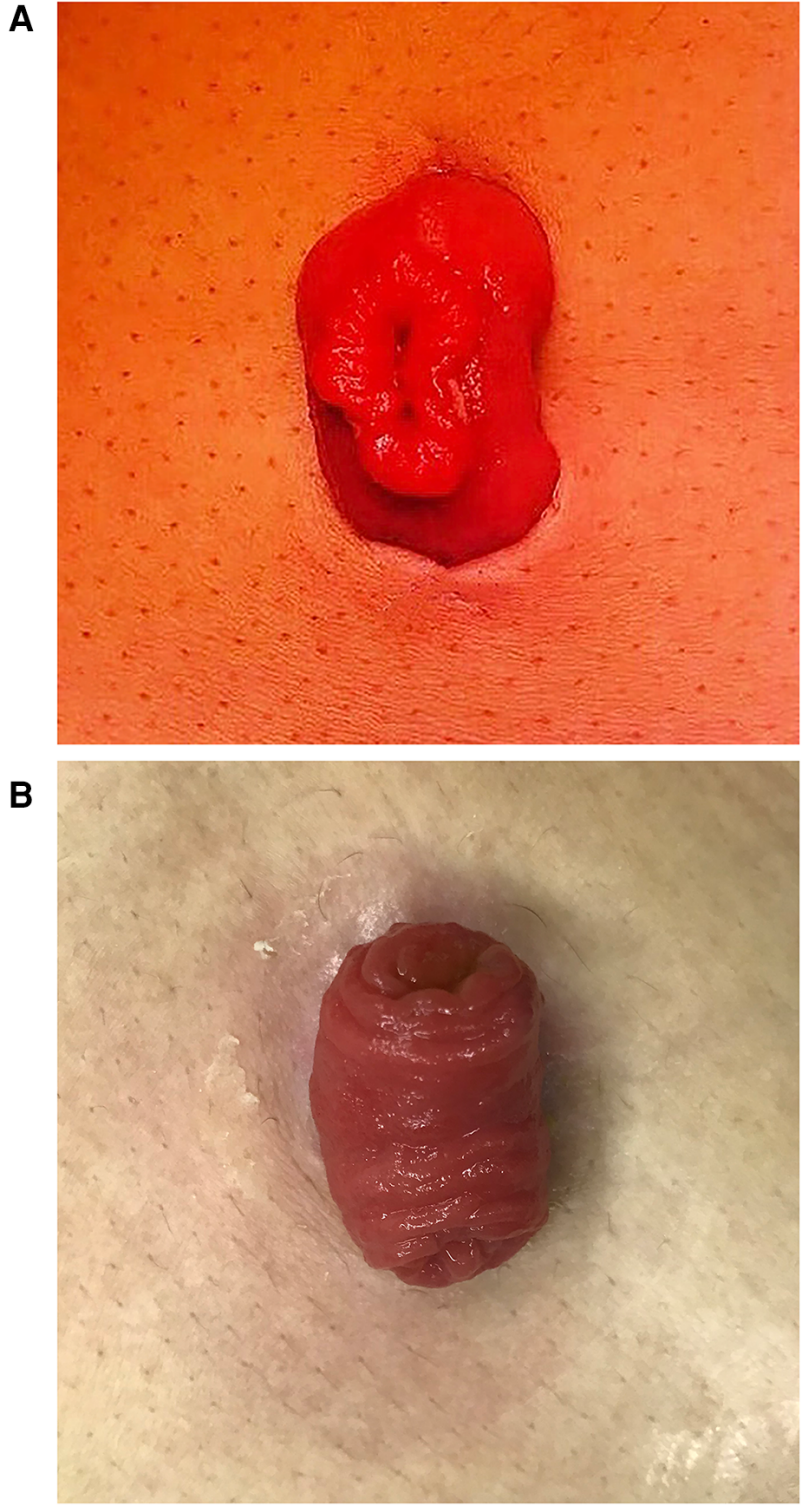
(**A**) The intestinal wall was incised longitudinally about 1–2 cm in length. (**B**) The intestinal wall of the stoma naturally turned over (1 month after surgery).

The terminal ileum approximately 25–30 cm proximal to the ileocecal junction was exteriorized through the stoma incision and rotated 180° clockwise to ensure that the proximal limb was at the caudal side. A 3-0 polyglactin 910 suture (VCPB864D, Coated VICRYL Plus, Ethicon, United States) was used to sew into the skin 0.5–1.0 cm away from the incision midpoint at one side of the incision (Figure 2A). Then, the suture was passed through the avascular area of the ileal mesentery (Figure 2B) and subsequently sewed out from the opposite side of the incision at an equal distance (Figure 2C). Finally, the suture was passed through the avascular area of the mesentery to the original side (Figure 2D). The suture thread was tightened and knotted to fix the ileal stoma (Figure 2E). The intestinal wall was incised longitudinally about 1–2 cm in length (Figure 3A). The intestinal wall of the stoma would naturally turn over (Figure 3B).

In the TM group, laparoscopic low anterior resection with end-to-end anastomosis was performed with a 5 cm abdominal midline incision for specimen extraction. An appropriate matching incision was established in the right lower quadrant. The seromuscular layer of the ileum was sutured intermittently in a circle with the peritoneum and the anterior rectal sheath using 3-0 polyglactin 910 suture, after which a plastic rod was placed as an ostomy bridge under the loop, half of the circumference of the antimesenteric portion of the bowel was opened, and the cut edges of the bowel were everted and fixed to the skin interrupted. After suturing the mucosal layer of the ileum transcutaneously, a 2–2.5 cm protrusion of the proximal limb was approximately achieved, and the stoma bag was placed.

### Statistical methods

The data were presented as a number for categorical variables and the mean ± SD for continuous variables. Categorical variables were compared by using Chi-square analysis or Fisher's exact test, and continuous variables were compared by using Student's *t*-test. All *P* values were two-sided, and a *P* value less than 0.05 indicated a significant difference. All data analysis was performed using SPSS software version 22.0 (IBM Corp).

## Results

A total of 70 patients met the inclusion criteria, including 30 patients who underwent PO and 40 patients who underwent the traditional procedure. No statistically significant differences were reported between the two groups in terms of preoperative clinical characteristics, such as sex, age, BMI, tumor distance from the dentate line, diabetes mellitus, and ASA grade. No significant differences in intraoperative blood loss, postoperative tumor size, and tumor pathological stage were found between the two groups. The details were shown in Table 1.

**Table 1:** Clinical and pathological characteristics of patients according to the technique.

Variables	PO (*n* = 30)	TM (*n* = 40)	*P* value
Sex			n.s.
Male	13	22	
Female	17	18	
Age (year)	60.1 ± 9.0	63.0 ± 10.4	n.s.
BMI (kg/m^2^)	24.0 ± 3.2	24.5 ± 3.5	n.s.
Diabetes mellitus	2	5	n.s.
ASA			n.s.
I–II	29	36	
III	1	4	
Distance from the dentate line (cm)	3.1 ± 1.3	3.6 ± 1.1	n.s.
Tumor size (cm)	3.2 ± 0.9	3.2 ± 1.0	n.s.
Pathologic T stage			n.s.
T1–T2	19	18	
T3	11	22	
Pathologic N stage			n.s.
N0	16	25	
N1–N2	14	15	

PO, prolapsing technique and one-stitch method of ileostomy; TM, traditional method; BMI, body mass index; ASA, American Society of Anesthesiologists grade.

The quality of the TME specimen was comparable in both groups. Two groups had no cases of positive circumferential resection margin. No patients with positive distal resection margin were identified in the PO group but four in the TM group, but the difference was not significant. The PO group had a shorter total operative time than the TM group (197.8 ± 43.4 vs. 218.3 ± 40.6 min, *P* = 0.047). The time of intestine function recovery in the PO group was shorter than that in the TM group (24.6 ± 3.8 vs. 32.7 ± 5.4 h, *P* < 0.001). Compared with the TM group, the average VAS score was significantly lower in the PO group (*P* < 0.001). Furthermore, anastomotic leakage (AL) occurred in six patients of the TM group, all of them were cured with conservative treatment. The incidence of anastomotic leakage in the PO group was significantly lower than that in the TM group (*P* = 0.034). The details are shown in Table 2.

**Table 2:** Perioperative outcomes of patients according to the technique.

Variables	PO (*n* = 30)	TM (*n* = 40)	*P* value
Total operative time (cm)	197.8 ± 43.4	218.3 ± 40.6	0.047
Blood loss (ml)	45.7 ± 28.6	43.6 ± 34.5	n.s.
Time to first flatus (h)	24.6 ± 3.8	32.7 ± 5.4	<0.001
Postoperative VAS scores			
First day	3.4 ± 0.9	4.4 ± 1.0	<0.001
Third day	1.9 ± 0.7	3.0 ± 0.8	<0.001
Quality of TME			n.s.
Complete	27	36	
Nearly complete	2	3	
Incomplete	1	1	
Anastomotic leakage	0	6	0.034
CRM (+)	0	0	n.s.
Distal margin (+)	0	3	n.s.

PO, prolapsing technique and one-stitch method of ileostomy; TM, traditional method; VAS, visual analog scale; TME, total mesorectal excision; CRM, circumferential resection margin.

The operative time of loop ileostomy was 2.0 ± 0.6 min in the PO group, which was significantly less than 15.1 ± 2.9 min in the TM group. Skin irritation was observed in 2 patients in the PO group and 10 patients in the TM group, and there was a significant difference (*P* = 0.044). No differences were found between the two groups when considering other stoma-related complications such as necrosis, bleeding, stricture, retraction, prolapse, mucocutaneous separation, and parastomal hernia (Table 3).

**Table 3:** Surgical outcomes and the stoma-related complications according to the technique.

Variables	PO (*n* = 30)	TM (*n* = 40)	*P* value
Operative time (min)	2.0 ± 0.6	15.1 ± 2.9	<0.001
Stoma necrosis	0	1	n.s.
Stoma bleeding	0	1	n.s.
Stoma stricture	3	3	n.s.
Stoma prolapse	1	2	n.s.
Stoma retraction	1	1	n.s.
Mucocutaneous separation	0	3	n.s.
Skin irritation	2	10	0.044
Parastomal hernia	3	4	n.s.

PO, prolapsing technique and one-stitch method of ileostomy; TM, traditional method.

## Discussion

Since NOSES was first reported by Franklin et al. (5) in 1993, a variety of new minimally invasive surgical techniques have been emerging. Based on the tumor location and the method for specimen extraction, Guan et al. (3) proposed practical NOSES techniques, including laparoscopic radical resection of low rectal cancer by the transanal prolapsing technique. Since there is no auxiliary incision in the abdominal wall and the abdominal cavity is not exposed, NOSES has a better minimally invasive surgical effects such as less postoperative pain, faster recovery of bowel function, and better cosmetic effects. The absence of auxiliary incision that needs to be sutured can reduce the operation time. Additionally, the prolapsing technique overcomes the difficulties in determining the precise transection line and transection of the rectum close to the anal sphincter in a narrow pelvic space. The everted rectum is transected under direct vision, avoiding multiple-stapler firings and reducing the risk of positive surgical margin.

One of the key technical difficulties of NOSES is the placement of the anvil in the proximal colon. The anvil can be usually inserted into the proximal colon through the rectum. Some surgeons also inserted the anvil into the abdominal cavity via the extended port hole (6) in the right lower quadrant. It is hard to fix the anvil to the proximal colon under total laparoscopy, as excellent skills of operation and team cooperation ability are required. At present, there are many kinds of fixation methods, such as extrusion (3), reverse puncture (7), snare ligation (8), and manual purse-string suture (9). Each method has its unique advantages and disadvantages. In this study, the modified anvil placement was extracorporeally performed by inserting the anvil after extraction of the proximal colon from the ileostomy incision, thus avoiding the risks of abdominal cavity contamination and tumor cell shedding or implantation metastasis during the process of placement of the anvil in the abdomen and fixation of it to the proximal colonic stump intracorporeally. In parallel, the purse-string suture method may lower the risk of anastomotic leakage caused by more intersections of staple lines and reduce the hospitalization costs owing to the reduced use of stapling cartridges. This technique is superior to the laparoscopic purse-string suture or snare ligation method in terms of simple operation and time savings. Moreover, compared with transanally pulling the proximal colon out, there is no need to fully mobilize the sigmoid colon or splenic flexure, which is more suitable for patients with short sigmoid colon or fatty mesosigmoid.

Anastomotic leakage remains the most severe complication of sphincter-preserving surgery for low rectal cancer and was reported to occur in about 10% (10) of patients after rectal cancer surgery. A systematic review and meta-analysis (11) including four RCTs and nine comparative studies with more than 2,000 patients showed that a diverting stoma may lower the rate of AL, additionally reducing the risk of reoperations significantly. The rate of diverting stoma after NOSES for low rectal cancer was even up to 77.2%–100% (6, 12). Patients who conform to the following criteria are encouraged to undergo protective ostomy: (1) patients with preoperative radiotherapy or intestinal obstruction; (2) the distance between the lower margin of the tumor and the anal verge <5 cm, especially <3 cm; and (3) poor patient's general status, including advanced age, malnutrition, hypoproteinemia, preoperative anemia, hypertension, diabetes, and other basic diseases associated with increased risk of AL (13). In this study, the new ostomy technique created a stoma, whose edge was in a shape of “8” via tightening the midpoints of the skin on both sides. The edge of the stoma was in complete contact with the intestinal wall that may reduce the occurrence of mucocutaneous separation. Furthermore, the continuous eversion of the intestinal mucosa after the opening of the stoma made the stoma protrude completely out from the skin in a “fungating” shape. The shape was “large at the top and small at the bottom,” which was beneficial to stoma care by using a smaller perforated stoma chassis. Because the ileum of the stoma was completely positioned in the bag, and the chassis could surround the bottom of the stoma closely, the one-stitch method could reduce the rate of peristomal skin disorders. The one-stitch method of ileostomy facilitated the postoperative care as there was no need of the supporting rod. As a result of the simplified suture process and reducing the number of stoma sutures, the operation time was shortened.

This study has some limitations. First, it was a retrospective single-institute analysis, and the sample size was small. Moreover, the current study design focused on the short-term efficacy, especially perioperative outcomes and stoma-related complications. Anal sphincter function was not preoperatively investigated, functional outcome was not well addressed because all study patients simultaneously underwent loop ileostomy at the time of rectal resection, and follow-up period was short. Third, we did not investigate oncologic outcomes. Due to the limitations of this study, the above conclusions need to be further validated by high-quality prospective controlled study with a large sample and long follow-up.

However, the rectal prolapsing technique is mainly applicable to patients with small tumor size. Routine protective ileostomy is not recommended only if there is a potentially high risk of AL. With the wide application of neoadjuvant chemoradiotherapy and the increase of patients at an early stage, the combination of rectal prolapsing technique and the one-stitch ileostomy could be an option for more cases.

## Conclusion

Integration of prolapsing technique and the one-stitch method of ileostomy during laparoscopic low anterior resection is safe and feasible, which can simplify the operation and reduce the technical difficulty. Therefore, it could be an alternative to laparoscopic low anterior resection without the advancement of any special instruments or skills.

## Data Availability

The raw data supporting the conclusions of this article will be made available by the authors, without undue reservation.
